# Supplementary material for: Integrated Analysis of the Lung Microbiome and Metabolome Reveals Associations Between Amino Acid Metabolism and Pulmonary Fibrosis in a Bleomycin-Induced Mouse Model
Source: Int J Mol Sci. 2026 Jun 30;27(13):5895. doi: 10.3390/ijms27135895 (PMC13362081; doi:10.3390/ijms27135895)
Supplement: Supplementary file 1 [file ijms-27-05895-s001.zip › result/5.MetKeggEnrichment/5-MetKeggEnrichment-readme.pdf]

MetKEGGEnrichment Readme

|-- 5.MetKEGGEnrichment 【差异代谢物 KEGG 富集分析结果目录】
| -- *.vs.* 【比较的样品对目录】
| |-- *.vs.*_{all}_KEGG_classification.{xls,xlsx}【差异代谢物 KEGG 分类分析列表】
| |-- *.vs.*_{all}_KEGG_classification.{png,pdf} 【差异代谢物 KEGG 分类图】
| |-- *.vs.*_{all}.kegg_enrichment.{xls,xlsx} 【差异代谢物 KEGG 富集结果列表】
| |-- *.vs.*_{all}.KEGG_Enrich.scatterplot.{png,pdf}【KEGG 富集气泡图】
| |-- *.vs.*_{all}_KEGG_map 【KEGG 通路图】
| |-- *.vs. *_{all}.KEGG_net.{png,pdf}【差异代谢物 KEGG 调控网络图。注： 如果 信息搜集表没有填写“KEGG 物种缩写”， 则无此结果】
| |-- *.vs. *_{all}.KEGG_net.xls 【差异代谢物 KEGG 调控网络结果列表。注：如果信息搜集表没有填写“KEGG 物种缩写”，则无此结果】
| |-- *.vs. *_{all}.KEGG_net_id_all.xls【差异代谢物 KEGG 调控网络互作关系列表 。注：如果信息搜集表没有填写“KEGG 物种缩写”，则无此结果】

***.vs.*_all.KEGG_classification.{xls,xlsx}**

第一列： KO_Pathway_Level1，富集到 KEGG PATHWAY 的一级分类条目；
第二列： KO_Pathway_Level2，富集的 KEGG PATHWAY 的二级分类条目；
第三列： Meta_Num，富集到 KEGG PATHWAY 的数目；
第四列： Metabolites，富集到 KEGG PATHWAY 的代谢物 ID；

***.vs.*_{all}.kegg_enrichment.{xls,xlsx}**

第一列： MapID，富集的 KEGG PATHWAY 的 ID；
第二列： MapTitle，富集的 KEGG PATHWAY 名称；
第三列： Pvalue，富集分析的 Pvalue；
第四列： x，与该通路相关的差异代谢物的数目；
第五列： y，与该通路相关的背景（所有）代谢物的数目；
第六列： n，KEGG 注释的差异代谢物数目；
第七列： N，KEGG 注释的背景（所有） 代谢物的数目；
第八列： EnrichDirect，富集指向,Over 代表富集；
第九列： MetaIDs，富集到的代谢物 list；
第十列： KEGG_cpd_id，KEGG 数据库代谢物的 ID；

***.vs. *_{all}.KEGG_net.xls**

第一列： KEGG.id，KEGG 调控网络每个节点的 ID；
第二列： Entry.type，KEGG 调控网络每个节点的类型；
第三列： KEGG.name，KEGG 调控网络每个节点的名称；
第四列： p.score，基于无效假设的分析的期望值和协方差矩阵的 z-score 计算得到得分值；

***.vs. *_{all}.KEGG_net_id_all.xls**

第一列： id，基于*.vs. *_all.KEGG_net.xls，列出 KEGG 调控网络互作关系第一个 ID；
第二列： id2，基于*.vs. *_all.KEGG_net.xls，列出 KEGG 调控网络互作关系第二个 ID；

***.vs.*_{all}.KEGG_Enrich.scatterplot.{png,pdf}**

KEGG 富集气泡图：根据 KEGG 富集结果，选取 P-value 值由小到大排序 Top20 的通路绘制气泡图。横坐标为 x/y（相应代谢通路中差异代谢物的数目/该通路中鉴定出总代谢物数目），值越大，表示该通路中差异代谢物富集程度越高，纵坐标为 KEGG 通路名称。点的颜色代表超几何检验的 P-value 值， 值越小，说明检验的可靠性越大、越具统计学意义。点的大小代表相应通路中差异代谢物的数目，越大，该通路内差异代谢物就越多。

***.vs. *_{all}.KEGG_net.{png,pdf}**

KEGG 调控网络图：基于前期分析得到的差异代谢物构建基于网络的富集分析。结果包括代谢通路、模块、酶、反应及代谢物。可以反映特定研究条件下代谢通路之间的交集以及靶向潜在的酶和代谢物，表明扰动如何在通路水平上传播以及通路如何相互影响，从而增强了结果的可解释性。在取得每组对比差异代谢物的匹配信息后，我们对对应物种的 KEGG 数据库进行通路搜索和调控互作网络分析。

***.vs.*_{all}_KEGG_map**

KEGG 通路图解释说明：

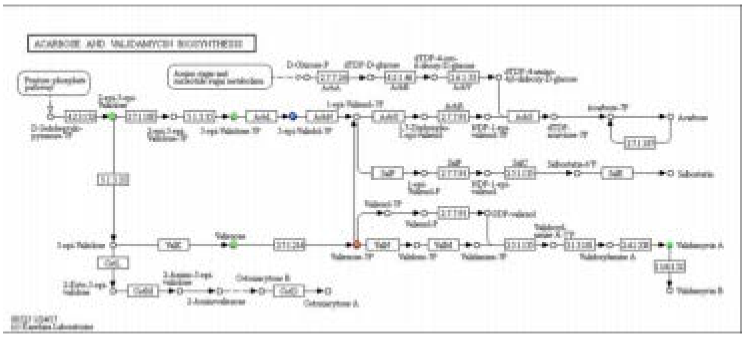

KEGG 富集通路图

KEGG PATHWAY Database（https://www.genome.jp/KEGG/pathway.html）是根据相关知识手绘的代谢通路数据库，目前分为以下几类：1.新陈代谢、 2.遗传信息加工、3.环境信息加工、4.细胞过程、 5.生物体系统、 6.人类疾病、7.药物开发

说明：

绿色：注释到的代谢物

蓝色框： 下调差异代谢物

红色框： 上调差异代谢物

黄色圆圈：同时包含上下调的代谢物

下方为图例解释，仅供参考；其他说明详见官网解释：

https://www.genome.jp/KEGG/document/help_pathway.html

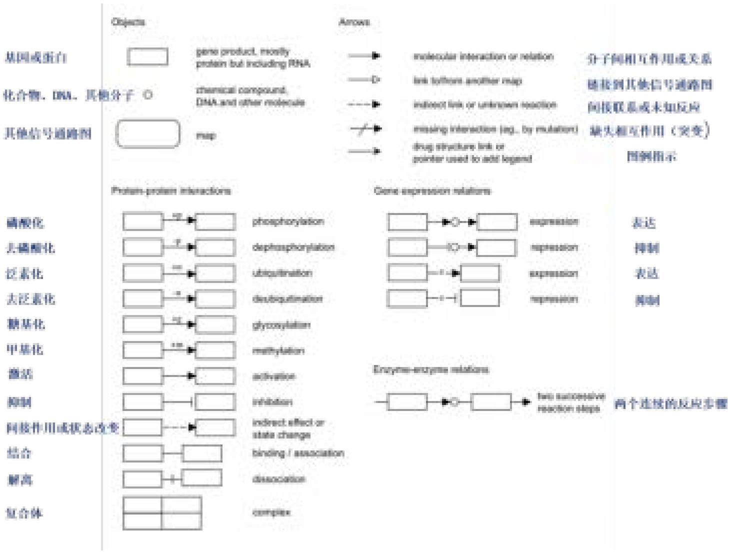
Objects
Arrows
基因或蛋白
gene product, mostly protein but including RNA
化合物、DNA、其他分子
chemical compound, DNA and other molecule
其他信号通路图
map
molecular interaction or relation
分子间相互作用或关系
link to/from another map
链接到其他信号通路图
indirect link or unknown reaction
间接联系或未知反应
missing interaction (eg., by mutation)
缺失相互作用（突变）
drug structure link or pointer used to add legend
图例指示
Protein-protein interactions
磷酸化
phosphorylation
去磷酸化
dephosphorylation
泛素化
ubiquitination
去泛素化
deubiquitination
糖基化
glycosylation
甲基化
methylation
激活
activation
抑制
inhibition
间接作用或状态改变
indirect effect or state change
结合
binding / association
解离
dissociation
复合体
complex
Gene expression relations
expression
表达
repression
抑制
expression
表达
repression
抑制
Enzyme-enzyme relations
two successive reaction steps
两个连续的反应步骤
